# A 6-Year Longitudinal Study of Adolescents and Mothers Depression Symptoms and Their Perception of Support and Conflict

**DOI:** 10.1007/s10578-019-00952-y

**Published:** 2020-01-14

**Authors:** William W. Hale, Stefanie A. Nelemans, Wim H. J. Meeus, Susan J. T. Branje

**Affiliations:** Research Center Adolescent Development, Utrecht University, P.O. Box 80.140, 3508 TC Utrecht, The Netherlands

**Keywords:** Adolescent, Conflict, Depression, Mother, Interpersonal relationship, Support

## Abstract

Interpersonal theories of adolescent depression assume that adolescent and maternal depression symptoms affect adolescent and maternal perceptions of their relationship quality. However, little attention has been given to examining potential bidirectional prospective associations between both adolescent and maternal perceptions of the mother–adolescent relationship and adolescent and maternal depression symptoms across adolescence. We hypothesized that the longitudinal associations between adolescent and maternal depression symptoms and adolescent and maternal perception of conflict and support in the mother–adolescent relationship would be bidirectional. In this 6-year longitudinal study, 497 adolescents (*M*_age_ = 13.03) and their mothers participated. Each year both adolescents and their mothers completed questionnaires of their depression symptoms and their perception of conflict and support in the mother–adolescent relationship. Structural equation modelling cross-lagged panel models were constructed and analyzed. The cross-lagged panel models found bidirectional longitudinal associations between adolescent depressive symptoms and higher adolescent-reported conflict and lower adolescent-reported support. In contrast, maternal depressive symptoms were only unidirectionally associated with higher maternal-reported conflict, lower maternal-reported support and higher adolescent depression symptoms. Finally, positive bidirectional longitudinal associations were found between adolescent-reported and maternal-reported conflict, and between adolescent-reported and maternal-reported support. The findings of this study are discussed in relation to Interpersonal Psychotherapy for Depressed Adolescents (IPT-A).

## Introduction

In previous research it has been found that both adolescent depression and maternal depression symptoms are positively associated with one another and both are also negatively associated with adolescent and maternal perceived relationship quality [1, 2]. Additionally, adolescent and maternal perceived negative relationship quality, in turn, has found to be associated with adolescent depression symptoms [3]. This applies not only to conflict being a risk factor for increasing depression symptom severity in adolescents [4, 5], but also to support, which buffers against adolescent depression symptom initiation and depression symptom severity [6]. In the study of interpersonal theories of adolescent depression [7–9], perceived relationship quality between the adolescent and his/her mother has been examined and these studies have shown maternal depression to be positively associated with adolescent and maternal perceived conflict [10–13] and that maternal support buffers against the negative effects of depression symptoms [14–16]. Furthermore, several studies have shown that a mother’s depression symptom severity has been shown to strengthen her adolescent’s depression symptom severity [17, 18]. Taken together, these findings demonstrate that adolescent and maternal perceived relationship quality and adolescent and maternal depression symptoms have a significant association with one another. These findings of previous studies have important implications not only for our theoretical understanding of the associations between depression and perceived relationship quality in families, but also for clinical practice which focuses on improving perceived relationship quality in the treatment of adolescent depression, such as Interpersonal Psychotherapy for Depressed Adolescents (IPT-A; [19]).

Perceived relationship quality has an important impact on adolescent depression. As was noted in a review of the literature that focused on adolescent and mother depression in interpersonal relationships: “Although disturbed relationships between depressed persons and their families cannot be reduced to the dysfunction of the depressed person, focusing attention on the way depressed individuals interact with key people in their lives may shed some light on the way they affect and are affected by each other.” ([20] p. 397). They suggest that studies that focus on the interpersonal relationship between depressed adolescents and their mothers can help to identify maladaptive patterns which will advance our theoretic knowledge of interpersonal theories of adolescent depression. By studying perceived relationship quality (such as conflict and support) longitudinally, the patterns in which conflict and support affect adolescent depression maintenance can be seen. It is with this specific knowledge that inventions such as IPT-A will be able to develop and “offer specific techniques to modify maladaptive social behaviors [such as conflict] that maintain depression” (p. 403). Additionally, the buffering effects of support against adolescent depression can also be incorporated into inventions such as IPT-A. Moreover, as these same authors note, this knowledge ultimately informs the development of prevention programs for adolescent depression.

However, until now, little attention has been given to longitudinally examining both adolescent and maternal conflict and support with the same mother and adolescent cohort to be able to examine the prospective nature of the observed associations with adolescent and maternal depression symptoms. Additionally, previous perceived relationship quality studies have focused on either support or conflict, but not both, and generally only measured either the adolescent’s depression symptoms or the mother’s depression symptoms. The only study that examined both mother and adolescent judgements of support and conflict in their interpersonal relationship in relation to adolescent depression symptoms was conducted by Sheeber et al. [21]. This study [21] found that conflict was positively associated, and support negatively associated, with adolescent depression symptoms at baseline and a year later.

We should also note that maternal depression symptoms were not included in the Sheeber study [21]. Therefore, in this study, we will go further by also including maternal depression symptoms. Additionally, the Sheeber study only focused on one year in an adolescent’s life in a diverse age group ranging from 14 to 20 years of age. In this study we have employed a more homogenic group that is primary composed of 11 to 12-year-olds and followed them for a 6-year period (until they were 17 to 18 years of age respectively). Finally, the Sheeber study combined both the adolescent’s and mother’s judgement of conflict into one latent variable; the same was also done for support. However, we examined the adolescent’s and mother’s judgement of conflict as individual variables to examine what their individual associations are with maternal depression symptoms and adolescent depression symptoms, respectively; the same was also done for support.

In light of the findings of the aforementioned studies, we hypothesized that adolescent and maternal conflict have positive longitudinal associations with adolescent and maternal depression symptoms, whereas adolescent and maternal support have negative longitudinal associations with adolescent and maternal depression symptoms. Furthermore, we also hypothesized that adolescent and maternal depression symptoms have positive longitudinal associations with adolescent and maternal conflict, and adolescent and maternal support have negative longitudinal associations with adolescent and maternal support. In other words, we hypothesized that adolescent and maternal perceived conflict, and adolescent and maternal depression symptoms will have a bidirectional and positive association with one another. Additionally, we hypothesized that adolescent and maternal perceived support, and adolescent and maternal depression symptoms will have a bidirectional and negative association with one another.

In this study, we used structural equation modeling (SEM) to be able to examine the longitudinal associations between adolescent and maternal depression symptoms, adolescent-reported quality of the mother–adolescent relationship, and maternal-reported quality of the mother–adolescent relationship in one and the same statistical model. Specifically, we tested separate models for negative and positive aspects of relationship quality: one for conflict and one for support. The reason for analyzing two models, and not one latent factor model comprised of both conflict and support, is because previous studies have demonstrated that support and conflict are two distinct aspects of relationship quality and not simply one construct with a continuum ranging from support to conflict [22, 23]. Finally, in our models we included adolescent gender as a potential covariate. The reason for doing this is because depression prevalence rates are higher among female adolescents than among males [24].

In summary, we hypothesized that adolescent and maternal perceived conflict and adolescent and maternal depression symptoms will have bidirectional and positive associations with one another. Additionally, we hypothesized that adolescent and maternal perceived support and adolescent and maternal depression symptoms will have bidirectional and negative associations with one another.

## Method

### Participants

Participants in this 6-year longitudinal community study were 497 adolescents (57% boys; 96% were either 12 or 13 years of age at T_1_; *M*_age_ T_1_ = 13.03 years, *SD*_age_ T_1_ = 0.46) and their mothers (*M*_age_ T_1_ = 44.41, *SD*_age_ T_1_ = 4.45). Participants were part of the ongoing RADAR-Y (Research on Adolescent Development and Relationships-Young cohort) study [25]. All adolescents identified themselves as ethnic Dutch and attended the first year of high school at the start of the study. The majority of adolescents lived in intact two-parent families (85.2%) at the start of the study. Furthermore, based on parents’ job level, 10.8% of the families were characterized by low SES.

Sample attrition was low over time, with 426 adolescents (85.7% of original sample of 497) and 420 mothers (84.5% of original sample of 497) still participating at the sixth annual measurement wave. Older adolescents at T_1_, *F*(1, 495) = 5.90, *p* = 0.02, partial η^2^ = 0.01, and adolescents from mothers with higher levels of maternal depression symptoms at T_1_, *F*(1, 487) = 6.16, *p* = 0.01, partial η^2^ = 0.01, were more likely to drop out of the study, but there were no significant differences between adolescents participating at all six measurement waves and those dropping out of the study in terms of gender, χ^2^(1) = 0.79, *p* = 0.38, and adolescent depression symptoms at T_1,_ adolescent-reported conflict and support in the perceived relationship quality of the mother–adolescent relationship at T_1_, and mother-reported conflict and support in the perceived relationship quality of the mother–adolescent relationship at T_1_, *F*(5, 481) = 1.40, *p* = 0.22. Little’s Missing Completely at Random test showed a normed χ^2^ (χ^2^/df; [26]) of 1.12, suggesting a good fit between sample scores with and without imputation. Missing data were handled in M*plus* with Full Information Maximum Likelihood (FIML [27]).

### Procedure

Participants were recruited from randomly selected primary schools in the western and central regions of the Netherlands (for a complete description of the sample, selection procedure, and the inclusion and exclusion criteria, please see [25])*.* Before the start of the study, adolescents and their mothers received written information about the research and provided written informed consent in order to participate. Each year, the adolescents and their mothers completed questionnaires during home-visits. Trained research assistants provided verbal instructions, given just prior to the filling in of the questionnaires to compliment the written instructions printed above each questionnaire. Other research assistants conducted the data entry to ensure that the data remained anonymous. This study was approved by the medical ethics committee of University Medical Center Utrecht (The Netherlands).

### Measures

#### Adolescent Depression Symptoms

We used the Reynolds Adolescent Depression Scale, second edition (RADS-2 [28]) to assess adolescent depression symptoms. The RADS-2 is a self-report questionnaire that consists of 23 items of depression symptoms that a person generally feels, which are measured on a 4-point scale ranging from 1 (*almost never*) to 4 (*usually*). Sample items include “I am sad” and “I feel like crying”. In a previous study the validity and reliability of this instrument have been shown to be good [29]. In this study, internal consistency for the total depression scale was found to be good across waves, with Cronbach’s α = 0.93 − 0.95.

#### Maternal Depression Symptoms

We used the adult depression subscale of the Adult Self-Report of the Achenbach System of Empirically Based Assessment (ASEBA; [30]) to assess maternal depression symptoms in the past six months. The ASEBA consists of 18 items measured on a 3-point scale ranging from 0 (*not true*) to 2 (*very true or often true*). Sample items include “I feel worthless or inferior” and “I am unhappy, sad, or depressed”. The validity and reliability of this instrument have been shown to be good in previous studies [31]. In this study, internal consistency for the total depression scale was found to be good across waves, with Cronbach’s α = 0.88 − 0.90.

#### Conflict

The 6-item negative interaction subscale of the Dutch version of the Network of Relationships Inventory-short version (NRI; [23]) was used to assess both adolescent-reported conflict and mother-reported conflict in the perceived relationship quality of the mother–adolescent relationship. All items were rated on a 5-point scale, ranging from 1 (*little or none*) to 5 (*the most*). Sample items include “How much do you and your mother argue with each other?” (for the adolescent) and “How much do you and your child argue with each other?” (for the mother). The psychometric properties of this instrument have been shown to be good [32]. In this study, internal consistency for the negative interaction subscale was found to be good for both the adolescent-reported scores, Cronbach α = 0.90 − 0.95, and the mother-reported scores, Cronbach α = 0.90 − 0.92.

#### Support

This study used the 8-item support subscale of the Dutch version of the NRI [23] to assess both adolescent-reported support and mother-reported support in the perceived relationship quality of the mother–adolescent relationship. All items were rated on a 5-point scale, ranging from 1 (*little or none*) to 5 (*the most*). Sample items include “How much does your mother really care about you?” (for the adolescent) and “How much do you really care about your child?” (for the mother). The psychometric properties of this instrument have been shown to be good [32]. In this study, internal consistency for the support subscale was found to be acceptable for both the adolescent-reported scores, Cronbach α = 0.78 − 0.85, and the mother-reported scores, Cronbach α = 0.71 − 0.78.

### Statistical Analyses

To address our study aims, we constructed two separate six-wave longitudinal cross-lagged panel models in M*plus* Version 7.4 [27]. Specifically, two models were constructed. One model included adolescent-reported conflict and maternal-reported conflict associated with adolescent depression symptoms and maternal depression symptoms in a bidirectional fashion across adolescence. The second model included adolescent-reported support and maternal-reported support associated with adolescent depression symptoms and maternal depression symptoms in a bidirectional fashion across adolescence. All models included 1-year and 2-year stability paths (needed for satisfactory model fit) for all constructs across waves and all within-wave associations between all constructs over waves (i.e., T_1_ correlations and correlated change). Furthermore, the following bidirectional 1-year theoretically relevant cross-lagged paths were included in each model; the cross-lagged paths between adolescent-reported relationship quality and maternal-reported relationship quality across waves, the cross-lagged paths between adolescent depression symptoms and adolescent-reported relationship quality across waves, the cross-lagged paths between maternal depression symptoms and maternal-reported relationship quality across waves, and the cross-lagged paths between adolescent depression symptoms and maternal depression symptoms across waves. In addition, we included adolescent gender as potential covariate in all models by regressing all variables on adolescent gender.

Maximum likelihood estimation with standard errors and chi square robust to non-normality (MLR; [27]) was used in all models. Model fit was assessed with the Comparative Fit Index (CFI), the Standardized Root Mean Square Residual (SRMR), and the Root Mean Square Error of Approximation (RMSEA) and its 90% confidence interval (90% CI) using conventional standards [33]. In our baseline models, all longitudinal parameters were constrained to be time invariant (i.e., constrained to be equal across time) for reasons of parsimony. We compared our baseline models to more complex versions of these models in which our parameters of interest, the cross-lagged paths, were estimated freely across time. The comparative fit of models was tested with Satorra–Bentler scaled chi-square difference tests (ΔSBχ^2^; [34]).

## Results

### Descriptive Statistics

The means, standard deviations and intercorrelations between all T_1_ variables can be found in Table 1.

**Table 1:** Means and standard deviations of all variables and intercorrelations between all variables at T_1_

	1	2	3	4	5	6
1. Adolescent depression symptoms	–					
2. Maternal depression symptoms	0.22**	–				
3. Adolescent-reported support	− 0.29**	− 0.10*	–			
4. Mother-reported support	− 0.12**	− 0.11*	0.26**	–		
5. Adolescent-reported conflict	0.33**	0.17**	− 0.33**	− 0.13**	–	
6. Mother-reported conflict	0.26**	0.22**	− 0.14**	− 0.18**	0.45**	–
*M* (*SD*)	1.63 (0.49)	0.25 (0.27)	3.90 (0.53)	3.50 (0.43)	1.66 (0.58)	1.52 (0.53)

This data for T_2_–T_6_ can be requested from the first author

*p < 0.05

**p < 0.01

### Conflict and Depression Symptoms

**Fig. 1:**
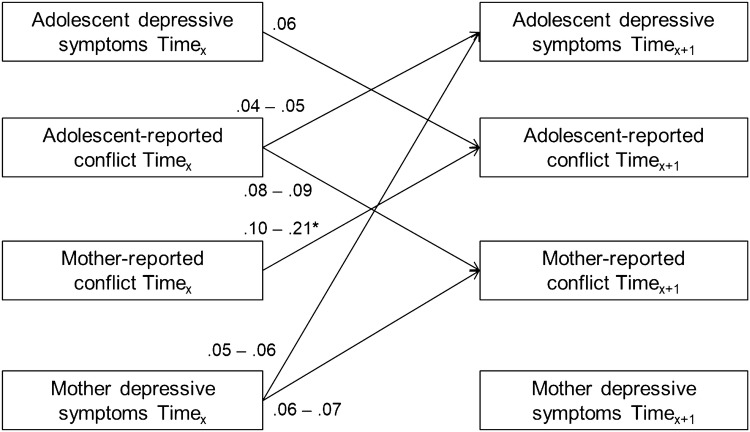
Overview of all significant (*p* < 0.05) standardized cross-lagged associations (βs) in the model concerning adolescent and maternal depression symptoms and adolescent and maternal reported conflict across six successive years. *Longitudinal cross-lagged paths from mother-reported conflict to adolescent-reported conflict were only significant from T_1_ to T_2_ and from T_2_ to T_3_

In analyzing adolescent-reported and maternal-reported conflict in the mother–adolescent relationship in association with adolescent depression symptoms and maternal depression symptoms, our fully constrained baseline cross-lagged panel model had a good fit to the data, SBχ^2^(268) = 424.28, CFI = 0.97, RMSEA [90%] = 0.03 [0.03 − 0.04], SRMR = 0.07. Freeing longitudinal cross-lagged paths from maternal-reported conflict to adolescent-reported conflict across time significantly improved the model fit, ΔSBχ^2^(4) = 11.14, *p* = 0.03, but stepwise freeing all other cross-lagged paths did not significantly improve the model fit, ΔSBχ^2^s(4) = 1.65 − 7.93, *p*s = 0.09 − 0.80. Hence, we kept all parameters in our model constrained to be time invariant except for longitudinal cross-lagged paths from maternal-reported conflict to adolescent-reported conflict, SBχ^2^(264) = 411.81, CFI = 0.97, RMSEA [90%] = 0.03 [0.03 − 0.04], SRMR = 0.06. An overview of all significant cross-lagged associations in our model is shown in Fig. 1.^1^

Results suggested two bidirectional longitudinal associations between adolescent depression symptoms, adolescent-reported conflict, and maternal-reported conflict. First, results showed a positive longitudinal association from adolescent-reported conflict to maternal-reported conflict across adolescence (βs = 0.08 − 0.09, *p*s < 0.001). We also found a positive longitudinal association from maternal-reported conflict to adolescent-reported conflict from early to mid-adolescence (T_1_ − T_3_, βs = 0.10 − 0.21, *p*s =  < 0.001 − 0.02). Second, results showed a positive longitudinal association from adolescent-reported conflict to adolescent depression symptoms (βs = 0.04 − 0.05, ps < 0.006). We also found a positive longitudinal association from adolescent depression symptoms to adolescent-reported conflict (βs = 0.06, *p*s < 0.001). Furthermore, a unidirectional positive longitudinal association was found from maternal depression symptoms to maternal-reported conflict (βs = 0.06 − 0.07, *p*s < 0.001). Finally, results showed a unidirectional positive longitudinal association from maternal depression symptoms to adolescent depression symptoms (βs = 0.05 − 0.06, *p*s = 0.003).

### Support and Depression Symptoms

**Fig. 2:**
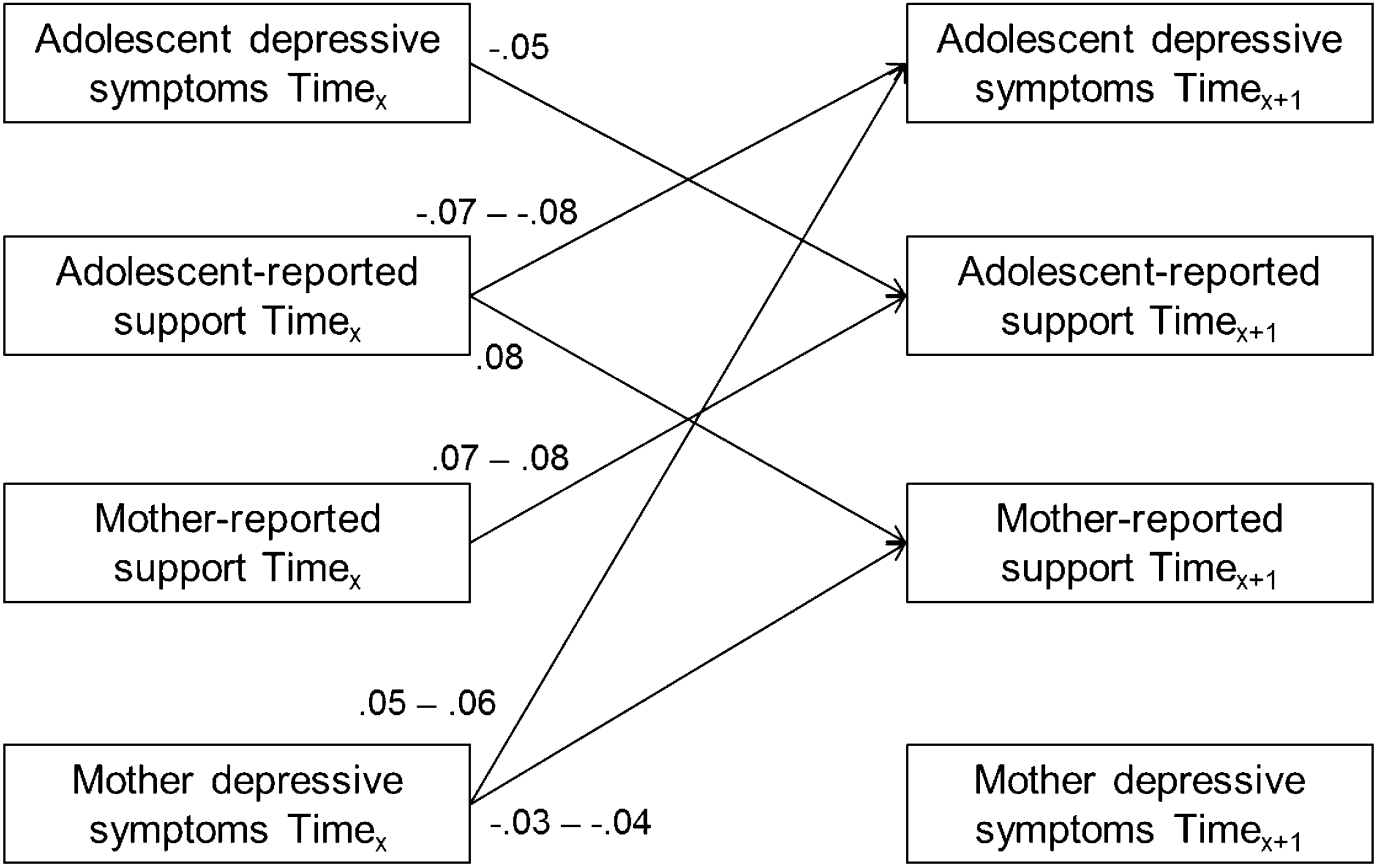
Overview of all significant (*p* < 0.05) standardized cross-lagged associations (βs) in the model concerning adolescent and maternal depression symptoms and adolescent and maternal reported support across six successive years

In analyzing adolescent-reported and maternal-reported support in the mother–adolescent relationship in association with adolescent depression symptoms and maternal depression symptoms, our fully constrained baseline cross-lagged panel model showed good fit to the data (SBχ^2^(268) = 463.73, CFI = 0.97, RMSEA [90%] = 0.04 [0.03 − 0.04], SRMR = 0.06). Stepwise freeing all cross-lagged paths did not significantly improve the model fit (ΔSBχ^2^s(4) = 0.85 − 9.51, *p*s = 0.05 − 0.93). Hence, we kept all parameters in our model constrained to be time invariant. An overview of all significant cross-lagged associations in this model is shown in Fig. 2.

Results suggested a similar pattern of findings for support in the mother–adolescent relationship as for conflict in the mother–adolescent relationship. Specifically, results again suggested two bidirectional longitudinal associations between adolescent depression symptoms, adolescent-reported support, and maternal-reported support. First, results showed a positive longitudinal association from adolescent-reported support to maternal-reported support across adolescence (βs = 0.08, *p*s < 0.001). We also found a positive longitudinal association from maternal-reported support to adolescent-reported support (βs = 0.07 − 0.08, *p*s =  < 0.001). Second, results showed a negative longitudinal association from adolescent-reported support to adolescent depression symptoms (βs = − 0.07 to − 0.08, ps < 0.001). We also found a negative longitudinal association from adolescent depression symptoms to adolescent-reported support (βs = − 0.05, *p*s = 0.004). Furthermore, a unidirectional negative longitudinal association was found from maternal depression symptoms to maternal-reported support (βs = − 0.03 to − 0.04, *p*s = 0.007). Finally, results showed a unidirectional positive longitudinal association from maternal depression symptoms to adolescent depression symptoms (βs = 0.05 − 0.06, *p*s = 0.001).

## Discussion

In this study we hypothesized that adolescent-reported and maternal-reported perceived conflict in the mother–adolescent relationship and adolescent and maternal depression symptoms would have bidirectional and positive effects with one another. Additionally, we hypothesized that adolescent-reported and maternal-reported perceived support in the mother–adolescent relationship and adolescent and maternal depression symptoms would have bidirectional and negative effects with one another. This study found only partial support for our hypotheses: higher adolescent depressive symptoms were indeed bidirectionally associated longitudinally with higher adolescent-reported conflict and lower adolescent-reported support. However, higher maternal depressive symptoms were only unidirectionally associated with higher maternal-reported conflict, lower maternal-reported support and higher adolescent depression symptoms. Finally, positive bidirectional longitudinal associations were found between adolescent-reported and maternal-reported conflict, and between adolescent-reported and maternal-reported support.

In other words, there are bidirectional and positive longitudinal associations in how the adolescents and their mothers perceive either conflict or support. It would appear that the adolescent’s perception of conflict strengthens his/her mother’s perception of conflict and vice versa. The same can be said of support. Additionally, the only other finding of bidirectional and longitudinal associations was that of adolescent depression and adolescent perceived conflict and support. In respect to adolescent perceived conflict, the relationship with adolescent depression was positive, which would indicate that adolescent perceived conflict is a risk factor for adolescent depression. However, with respect to adolescent perceived support, the relationship with adolescent depression was negative, which would indicate that adolescent perceived support is a protective factor for adolescent depression.

The final two findings seem to indicate that a mother’s depression can be viewed as a psychosocial risk factor to the adolescent. The first finding seems to indicate that the mother’s depression negatively influences her perception of her relationship to her adolescent. And for the second finding it would appear that the mother’s depression negatively influences the adolescent’s depression. These findings agree with previous studies that have found that maternal depression acts as a significant risk factor for adolescent depression. In a review of the literature that focused on adolescent and mother depression in relation to interpersonal relationship [20], it was stated that depressed adolescent negative view of themselves strongly influenced by the negative evaluation they received from their mothers. In a similar review of the literature [35] it is asserted that studies have also found that depressed mothers many times tend to exhibit overcontrolling and overprotective behaviors toward their adolescent. These dysfunctional behaviors lead to conflict between the mother and adolescent and this conflict, in turn, leads to the adolescent developing depression symptoms [35].

The findings of this study emphasize the importance of including both mothers and adolescents in exploring how the perception of relationship quality affects one’s own depression symptoms [36]. These findings which are in line with interpersonal theories of depression might be also of interest to the interpersonal treatment of depression. Interpersonal theories of depression form the basis of IPT-A [19] which focuses on perceived relationship quality and depression. The findings of this present study would seem to emphasize the IPT-A, how an adolescent perceives the quality of his/her relationship with his/her mother influences the mother’s perception the quality of her relationship with her adolescent and vice versa. Additionally, the finding that maternal depressive symptoms influence adolescent depressive symptoms is also in line with interpersonal theories of depression and IPT-A. However, IPT-A generally only focuses on the adolescent [37], while this study demonstrates that the same interpersonal processes also occur in the mothers and that the mother’s depression negatively influences the adolescent’s depression. At the moment IPT-A is many times only given in an individual therapy form [37]. It is conceivable that the effects of IPT-A might be increased by also involving the mother in the therapy and exploring both the adolescent’s and the mother’s negative thoughts on their perception of the quality of their relationship. The associations found in this study could be addressed in future IPT-A effectiveness studies that include the adolescent’s mother in the therapy.

Nevertheless, an important limitation of this study should be acknowledged. This study focused only on self-reports of depression symptoms from adolescents and their mothers from the general community. The use of self-reports of depression symptoms from adolescents and their mothers from the general community should not be confused with a clinical diagnosis of a psychiatric disorder. A structured clinical interview could have been used to help determine the strength of the association between the self-reports of depression symptoms and an actual diagnosis. However, it has also been suggested that prospective longitudinal community studies of psychopathological symptom dimensions may help circumvent the problem of referral bias that frequently occurs in the clinical setting and may better characterize the course of developmental psychopathological symptoms [38]. Still, future studies in the clinical setting could be conducted to replicate these findings.

## Summary

In sum, interpersonal theories of adolescent depression assume that adolescent and maternal depression symptoms affect adolescent and maternal perceptions of their relationship quality. However, little attention has been given to examining potential bidirectional prospective associations between both adolescent and maternal perceptions of the mother–adolescent relationship and adolescent and maternal depression symptoms across adolescence. We hypothesized that the longitudinal associations between adolescent and maternal depression symptoms and adolescent and maternal perception of conflict and support in the mother–adolescent relationship would be bidirectional. In this 6-year longitudinal study, bidirectional longitudinal associations were found between adolescent depressive symptoms and higher adolescent-reported conflict and lower adolescent-reported support. In contrast, maternal depressive symptoms were only unidirectionally associated with higher maternal-reported conflict, lower maternal-reported support and higher adolescent depression symptoms. Finally, positive bidirectional longitudinal associations were found between adolescent-reported and maternal-reported conflict, and between adolescent-reported and maternal-reported support. The findings of this present study would seem to emphasize the interpersonal aspects of Psychotherapy for Depressed Adolescents (IPT-A), how an adolescent perceives the quality of his/her relationship with his/her mother influences the mother’s perception the quality of her relationship with her adolescent and vice versa. Additionally, the finding that maternal depressive symptoms influence adolescent depressive symptoms is also in line with interpersonal theories of depression and IPT-A. Generally, IPT-A only focuses on the adolescent, while this study demonstrates that the same interpersonal processes also occur in the mothers and that the mother’s depression negatively influences the adolescent’s depression. It is conceivable that the effects of IPT-A might be increased by also involving the mother in the therapy.

## Footnotes

1 In addition to all theoretically relevant 1-year cross-lagged paths, we added all other potential 1-year cross-lagged paths (i.e., the bidirectional 1-year cross-lagged paths between adolescent-reported relationship quality and maternal depression symptoms and between mother-reported relationship quality and adolescent depression symptoms) to our two final models to examine robustness of our findings. These models showed good fit to the data for both support, SBχ^2^(264) = 455.86, CFI = 0.97, RMSEA [90%] = 0.04 [0.03 − 0.04], SRMR = 0.06, and conflict, SBχ^2^(260) = 408.28, CFI = 0.97, RMSEA [90%] = 0.03 [0.03 − 0.04], SRMR = .06, but did not significantly improve the model fit for both, ΔSBχ^2^s(4) = 3.30 – 8.13, *p*s = 0.09 − 0.51. Importantly, including all possible 1-year cross-lagged paths in our models did not affect any of our findings concerning associations between adolescent-reported and mother-reported support and conflict in association with depression symptoms of both informants.

## References

[1] Hale WW, van der Valk I, Akse J, Meeus WHJ (2008). The interplay of early adolescent depressed mood, aggressive behavior and perceived parental rejection: a four year longitudinal community study. J Youth Adolesc.

[2] Withers MC, Cooper A, Rayburn AD, McWey LM (2016). Parent–adolescent relationship quality as a link in adolescent and maternal depression. Child Youth Serv Rev.

[3] Rabinowitz JA, Drabick DA, Reynolds MD (2016). Family conflict moderates the relation between negative mood and youth internalizing and externalizing symptoms. J Child Fam Stud.

[4] Hammen C, Brennan PA (2001). Depressed adolescents of depressed and nondepressed mothers: tests of an interpersonal impairment hypothesis. J Consult Clin Psychol.

[5] Young JF, Gallop R, Mufson L (2009). Mother–child conflict and its moderating effects on depression outcomes in a preventive intervention for adolescent depression. J Clin Child Adolesc Psychol.

[6] McWey LM, Claridge AM, Wojciak AS, Lettenberger-Klein CG (2015). Parent–adolescent relationship quality as an intervening variable on adolescent outcomes among families at risk: dyadic analyses. Fam Relat.

[7] Meeus W (2016). Adolescent psychosocial development: a review of longitudinal models and research. Dev Psychol.

[8] Klein DN, Torpey DC, Bufferd SJ, Beauchaine TP, Hinshaw SP (2008). Depression disorders. Child and adolescent psychopathology.

[9] O'Shea G, Spence SH, Donovan CL (2014). Interpersonal factors associated with depression in adolescents: are these consistent with theories underpinning interpersonal psychotherapy?. Clin Psychol Psychother.

[10] Castellani V, Pastorelli C, Eisenberg N, Caffo E, Forresi B, Gerbino M (2014). The development of perceived maternal hostile, aggressive conflict from adolescence to early adulthood: antecedents and outcomes. J Youth Adolesc.

[11] Hale WW, Keijsers L, Klimstra TA, Raaijmakers QAW, Hawk S, Branje SJT, Frijns T, Wijsbroek SA, Van Lier P, Meeus WH (2011). How does longitudinally measured maternal Expressed Emotion affect internalizing and externalizing symptoms of adolescents from the general community?. J Child Psychol Psychiatry.

[12] Sheeber LB, Davis B, Leve C, Hops H, Tildesley E (2007). Adolescents’ relationships with their mothers and fathers: Associations with depression disorder and subdiagnostic symptoms. J Abnorm Psychol.

[13] Vazsonyi AT, Belliston LM (2006). The cultural and developmental significance of parenting processes in adolescent anxiety and depression symptoms. J Youth Adolesc.

[14] Burke T, Sticca F, Perren S (2017). Everything’s gonna be alright! The longitudinal interplay among social support, peer victimization, and depression symptoms. J Youth Adolesc.

[15] Collishaw S, Hammerton G, Mahedy L, Sellers R, Owen MJ, Craddock N (2016). Mental health resilience in the adolescent offspring of parents with depression: a prospective longitudinal study. Lancet Psychiatry.

[16] Rueger SY, Malecki CK, Pyun Y, Aycock C, Coyle S (2016). A meta-analytic review of the association between perceived social support and depression in childhood and adolescence. Psychol Bull.

[17] Connell AM, Goodman SH (2002). The association between psychopathology in fathers versus mothers and children’s internalizing and externalizing behavior problems: a meta-analysis. Psychol Bull.

[18] Hammen C, Hazel NA, Brennan PA, Najman J (2012). Intergenerational transmission and continuity of stress and depression: depressed women and their offspring in 20 years of follow-up. Psychol Med.

[19] Mychailyszyn MP, Elson DM (2018). Working through the blues: a meta-analysis on interpersonal psychotherapy for depressed adolescents (IPT-A). Child Youth Serv Rev.

[20] Chiariello MA, Orvaschel H (1995). Patterns of parent–child communication: relationship to depression. Clin Psychol Rev.

[21] Sheeber LB, Hops H, Alpert A, Davis B, Andrews J (1997). Family support and conflict: prospective relations to adolescent depression. J Abnorm Psychol.

[22] Baumrind D, Lerner RM, Petersen AC, Brooks-Gunn J (1991). Parenting styles and adolescent development. Encyclopedia of adolescence.

[23] Furman W, Buhrmester D (1985). Children's perceptions of the personal relationships in their social networks. Dev Psychol.

[24] Hankin BL, Abramson LY, Moffitt TE, Silva PA, McGee R, Angell KE (1998). Development of depression from preadolescence to young adulthood: emerging gender differences in a 10-year longitudinal study. J Abnorm Psychol.

[25] Van Lier PAC, Frijns T, Neumann A, Den Exter Blokland E, Koot HM, Meeus W (2011) The RADAR Young study: design, description of sample, and validation of cohort assignment. Unpublished manuscript. https://easy.dans.knaw.nl/ui/datasets/id/easy-dataset:113721/tab/2

[26] Bollen K (1989). Structural equations with latent variables.

[27] Muthén LK, Muthén BO (1998–2015) Mplus user’s guide. Muthén & Muthén, Los Angeles

[28] Reynolds WM (2000). Reynolds adolescent depression scale-2nd edition (RADS-2).

[29] Osman A, Gutierrez PM, Bagge CL, Fang Q, Emmerich A (2010). Reynolds adolescent depression scale-second edition: a reliable and useful instrument. J Clin Psychol.

[30] Achenbach TM, Rescorla LA (2003). Manual for the ASEBA adult forms & profiles.

[31] Rescorla LA, Achenbach TM, Maruish ME (2004). The Achenbach System of Empirically Based Assessment (ASEBA) for Ages 18 to 90 Years. The use of psychological testing for treatment planning and outcomes assessment: Instruments for adults.

[32] Furman W, Buhrmester D (2009). Methods and measures: the network of relationships inventory: behavioral systems version. Int J Behav Dev.

[33] Hu L, Bentler PM (1999). Cutoff criteria for fit indexes in covariance structure analysis: conventional criteria versus new alternatives. Struct Equ Model.

[34] Satorra A, Bentler PM (2001). A scaled difference chi-square test statistic for moment structure analysis. Psychometrika.

[35] Stark KD, Banneyer KN, Wang LA, Arora P (2012). Child and adolescent depression in the family. Couple Family Psychol.

[36] Bogenschneider K, Pallock L (2008). Responsiveness in parent–adolescent relationships: are influences conditional? Does reporter matter?. J Marriage Fam.

[37] Spence SH, O’Shea G, Donovan CL (2016). Improvements in interpersonal functioning following interpersonal psychotherapy (IPT) with adolescents and their association with change in depression. Behav Cogn Psychother.

[38] Hale WW, Raaijmakers QAW, Muris P, Van Hoof A, Meeus WHJ (2009). One factor or two parallel processes? Comorbidity and development of adolescent anxiety and depression disorder symptoms. J Child Psychol Psychiatry.

